# Middle cerebral artery occlusion due to paradoxical embolism diagnosed by pulmonary artery thrombosis in the absence of deep vein thrombosis: A case report

**DOI:** 10.1016/j.radcr.2026.05.058

**Published:** 2026-06-03

**Authors:** Akinobu Ito, Hiroyuki Ikeda, Masakazu Okawa, Taichi Ikedo, Hideo Chihara, Shigeki Takada, Noritaka Sano, Takayuki Kikuchi, Chiaki Sakai, Yoshiki Arakawa

**Affiliations:** Department of Neurosurgery, Kyoto University Graduate School of Medicine, 54 Kawahara-cho, Shogoin, Sakyo-ku, Kyoto 606-8507, Japan

**Keywords:** Deep vein thrombosis, Paradoxical embolism, Pulmonary artery thrombosis, Thrombectomy

## Abstract

A definitive diagnosis of paradoxical embolism generally requires confirmation of an arterial embolic event, a right-to-left shunt, identification of a venous thrombus, and exclusion of other embolic sources. In clinical practice, pulmonary embolism may be identified even in the absence of detectable deep vein thrombosis. In the present case, paradoxical embolism was considered highly probable based on the identification of a pulmonary artery thrombus despite the absence of deep vein thrombosis. A 54-year-old woman underwent successful thrombectomy with subsequent neurological improvement. Repeated postoperative ultrasonography showed no evidence of deep vein thrombosis. Transesophageal echocardiography revealed a patent foramen ovale, and contrast-enhanced computed tomography identified a pulmonary artery thrombus in the absence of deep vein thrombosis. Anticoagulation therapy resulted in complete thrombus resolution without recurrence.

## Introduction

A definitive diagnosis of paradoxical embolism requires confirmation of an arterial embolic event, the presence of a right-to-left shunt, identification of a venous thrombus, and exclusion of other potential embolic sources [1]. Among venous thrombi, deep vein thrombosis is the most common, with more than 90% originating in the lower extremities [2,3]. Accordingly, when paradoxical embolism is suspected, evaluation for lower-extremity deep vein thrombosis is recommended as the initial diagnostic approach [4]. In contrast, establishing a diagnosis of paradoxical embolism based on the detection of a pulmonary artery thrombus in the absence of detectable deep vein thrombosis remains clinically challenging [5,6]. Here, we report a case in which paradoxical embolism was considered highly probable after mechanical thrombectomy for middle cerebral artery occlusion based on the identification of a pulmonary artery thrombus on contrast-enhanced computed tomography (CT), despite the absence of lower-extremity deep vein thrombosis.

## Case report

The patient was a 54-year-old right-handed woman with a medical history of transient ventricular tachycardia during pregnancy at the age of 40 years and a known nickel allergy. She had a 34-year smoking history of 10 cigarettes per day and no history of alcohol consumption. She was found collapsed at home and was transported to our hospital approximately 18 hours after the last known well time. Neurological examination revealed left-sided incomplete hemiparesis, dysarthria, motor aphasia, and sensory disturbances of the left upper and lower extremities, with a National Institutes of Health Stroke Scale score of 20. Electrocardiography showed no abnormalities. Blood pressure was 128/68 mmHg, and heart rate was 74 beats per minute with a regular rhythm. Based on a height of 158 cm and body weight of 56.6 kg, her body mass index was 22.67 kg/m². Laboratory testing showed a white blood cell count of 6,740/µL, hemoglobin level of 12.4 g/dL, platelet count of 323 × 10⁴/µL, C-reactive protein level of 0.09 mg/dL, brain natriuretic peptide level of 55.4 pg/mL, total cholesterol level of 225 mg/dL, high-density lipoprotein cholesterol level of 67 mg/dL, low-density lipoprotein cholesterol level of 148 mg/dL, triglyceride level of 73 mg/dL, hemoglobin A1c level of 5.9%, activated partial thromboplastin time of 26.3 seconds, prothrombin time–international normalized ratio of 0.97, and D-dimer level of 3.8 µg/mL. Noncontrast head CT showed no early ischemic changes (Alberta Stroke Program Early CT Score of 10), whereas a hyperdense sign was observed in the distal M1 segment of the right middle cerebral artery (Fig. 1A). Contrast-enhanced CT demonstrated occlusion of the distal M1 segment without evidence of an embolic source in the aortic arch or venous thrombosis within the scanned range; most of the pulmonary arteries were outside the field of view (Fig. 1B–D). Because approximately 18 hours had elapsed since the last known well time, intravenous thrombolysis was not performed, and mechanical thrombectomy was undertaken.

**Fig. 1:**
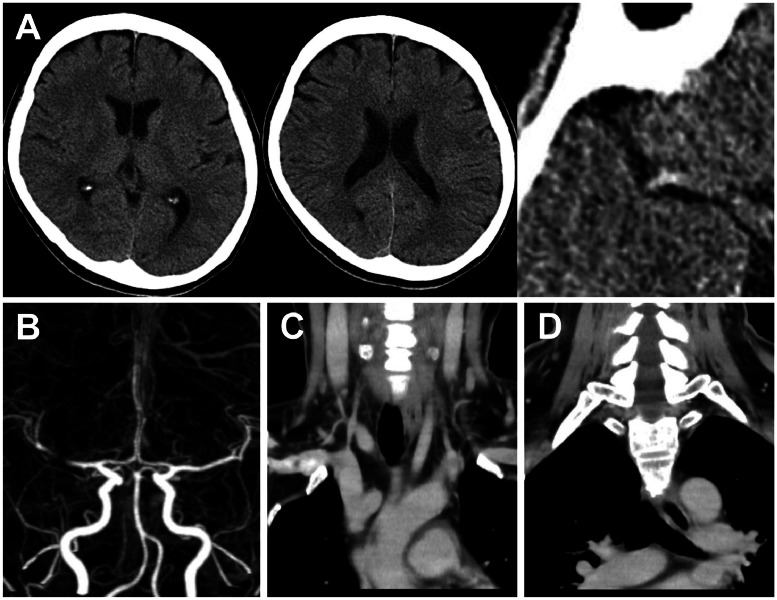
Preoperative imaging findings. (A) Head CT shows no early ischemic changes and a hyperdense sign in the right distal M1 segment. (B) CT angiography reveals occlusion of the right distal M1 segment and (C, D) Contrast-enhanced CT in the coronal view shows no embolic source in the aortic arch or its major branches, while the caudal portion of the pulmonary arteries is outside the field of view.

The procedure was initiated 54 minutes after hospital arrival. Cerebral angiography via a guiding catheter placed in the right internal carotid artery confirmed distal right M1 occlusion (Fig. 2A).

**Fig. 2:**
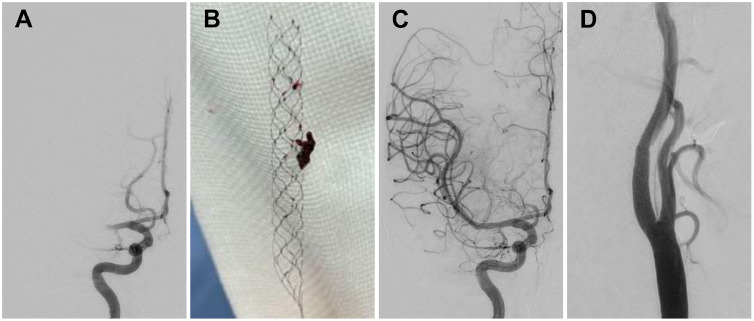
Intraoperative imaging findings. (A) Right internal carotid angiography before thrombectomy shows occlusion of the right M1 segment. (B) A red thrombus is retrieved using a stent retriever. (C) Right internal carotid angiography after thrombectomy shows recanalization of the right M1 segment and (D) Right common carotid angiography after thrombectomy shows no stenotic lesion in the cervical internal carotid artery.

A macroscopically red thrombus was retrieved using a combined technique involving an aspiration catheter and a stent retriever (Fig. 2B), and successful recanalization (modified Thrombolysis in Cerebral Infarction grade 2c) was achieved 18 minutes after procedure initiation (Fig. 2C). Right common carotid angiography revealed no abnormalities suggestive of an embolic source in the cervical internal carotid artery (Fig. 2D). The patient’s neurological deficits improved rapidly after the procedure, and postprocedural head CT showed no intracranial hemorrhage.

On day 2, diffusion-weighted magnetic resonance imaging (MRI) revealed a new cerebral infarction in the right lenticular nucleus (Fig. 3A and B). Magnetic resonance angiography showed sustained patency of the right M1 segment without restenosis or reocclusion (Fig. 3C). At this stage, the embolic source remained undetermined; therefore, antiplatelet therapy with aspirin 100 mg/day was initiated. A comprehensive evaluation for an embolic source was performed. Transthoracic echocardiography conducted by a cardiologist on day 2 showed no abnormalities, and lower-extremity venous ultrasonography revealed no thrombi in the bilateral femoral or popliteal veins. On day 3, repeat transthoracic echocardiography performed by a clinical laboratory technologist was also unremarkable, and venous ultrasonography demonstrated no thrombi from the inferior vena cava to the calf veins. The patient had no history of oral contraceptive use or hormone replacement therapy. Additional laboratory testing, including evaluations for coagulation disorders, thrombophilia, and autoimmune diseases, revealed no abnormalities. Continuous in-hospital cardiac monitoring and 24-hour Holter electrocardiography showed no arrhythmias.

**Fig. 3:**
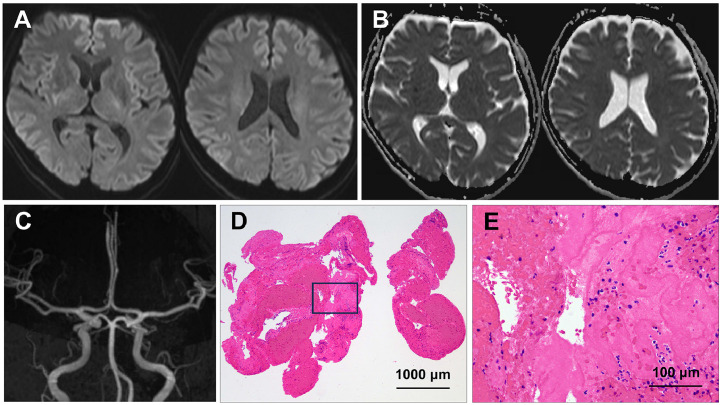
Postoperative radiological and histological findings. (A, B) Diffusion-weighted imaging (A) on day 2 shows a hyperintense lesion in the right lentiform nucleus with corresponding hypointensity on the apparent diffusion coefficient map (B). (C) Magnetic resonance angiography on day 2 shows sustained recanalization of the right M1 segment and (D, E) Histological examination of the thrombus reveals a mixed thrombus predominantly composed of fibrin and platelets containing red blood cells. Panel E shows a magnified view of the rectangular area in panel D.

Histopathological examination of the retrieved thrombus demonstrated a mixed thrombus predominantly composed of fibrin and platelets, with red blood cells accounting for approximately 40% of the thrombus volume (Fig. 3D and E). There was no evidence of endothelial cells, atherosclerotic plaque components, or infection.

On day 8, transesophageal echocardiography with a microbubble test demonstrated a grade 2 right-to-left shunt at rest, which increased to grade 4 during an adequate Valsalva maneuver (Fig. 4A) [7]. The patent foramen ovale had a tunnel length of 10.4 mm, an opening diameter of 2.1 mm, and a width of 13.4 mm, with demonstrable shunt flow on color Doppler imaging (Fig. 4B). The atrial septal excursion was 17 mm, consistent with an atrial septal aneurysm (Fig. 4C). No embolic sources were identified in the left atrial appendage, left atrium, or cardiac valves. On day 14, carotid MRI showed no high-intensity plaque on T1-weighted images in the right cervical internal carotid artery. On day 16, aortic MRI revealed no high-intensity plaques in the aortic arch on T1-weighted images; however, a high-intensity lesion suggestive of thrombus was observed in the right pulmonary artery (Fig. 5A and B). On the same day, contrast-enhanced CT of the trunk, including the lower extremities, demonstrated no evidence of deep vein thrombosis but revealed a contrast defect suggestive of thrombus in the right pulmonary artery (Fig. 5C and D). No findings suggestive of malignancy or infection were identified. Based on these findings, paradoxical cerebral embolism was considered highly probable. Aspirin was discontinued on day 17, and anticoagulation therapy with edoxaban 30 mg/day was initiated. The patient remained neurologically intact and was discharged home on day 23 with a modified Rankin Scale score of 0. Follow-up contrast-enhanced CT on day 37 confirmed complete resolution of the pulmonary artery thrombus, with no evidence of venous thrombosis (Fig. 5E). Because of her nickel allergy, percutaneous closure of the patent foramen ovale was not performed. Anticoagulation therapy has been continued, and no recurrent thromboembolic events have been observed during 6 months of follow-up.

**Fig. 4:**
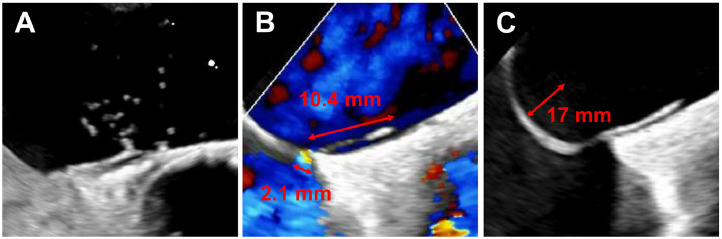
Postoperative transesophageal echocardiography findings. On day 8, a right-to-left shunt through a patent foramen ovale at rest is demonstrated by the microbubble test (A) and color Doppler imaging (B) and an atrial septal aneurysm is also observed (C).

**Fig. 5:**
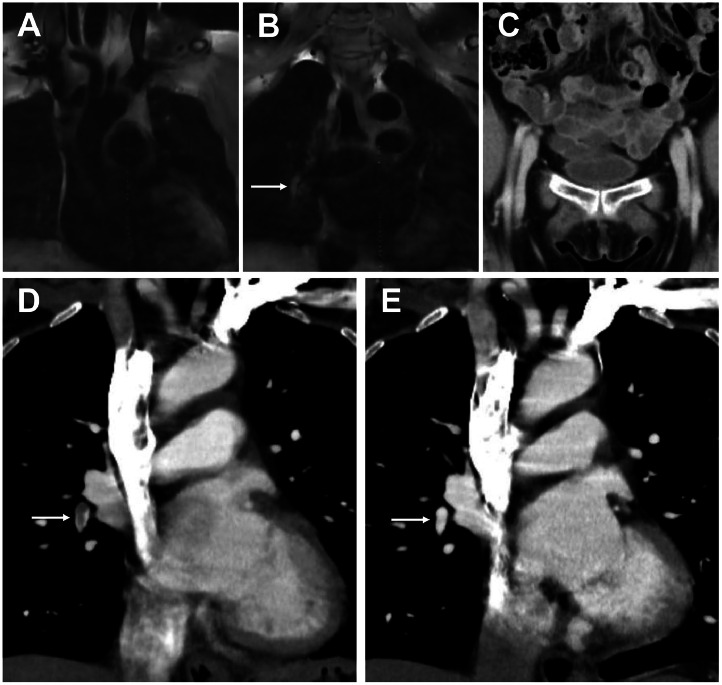
Imaging findings of the embolic source and pulmonary artery thrombus. (A, B) Chest T1-weighted MRI on day 16 shows no high-intensity plaque in the aortic arch and a hyperintense thrombus in the right pulmonary artery (arrow). (C, D) Contrast-enhanced CT of the trunk in the coronal view on day 16 shows a thrombus in the right pulmonary artery (arrow) without evidence of deep vein thrombosis and (E) Contrast-enhanced CT of the trunk in the coronal view on day 37 shows resolution of the right pulmonary artery thrombus (arrow).

## Discussion

This case involved a relatively young patient with embolic cerebral infarction and minimal atherosclerotic changes, in whom paradoxical embolism was considered a major differential diagnosis from the initial presentation. Lower-extremity venous ultrasonography was performed early to identify a potential venous thrombus as an embolic source; however, no deep vein thrombosis was detected. Although pulmonary embolism alone does not establish the embolic pathway or definitively prove paradoxical embolism, its presence in combination with a right-to-left shunt and the absence of other embolic sources strongly supported a diagnosis of probable paradoxical embolism in this case. Pulmonary embolism may increase right atrial pressure and facilitate right-to-left shunting through a patent foramen ovale; however, this represents a predisposing mechanism rather than direct diagnostic proof. Prevention of recurrent, potentially devastating cerebral embolism is critically important in patients undergoing mechanical thrombectomy. In the present case, a pulmonary artery thrombus was detected despite 15 days of antiplatelet therapy with aspirin, suggesting that antiplatelet therapy alone was insufficient. In contrast, the pulmonary artery thrombus resolved after initiation of anticoagulation therapy, underscoring the importance of selecting appropriate antithrombotic treatment according to the underlying pathophysiology. Randomized controlled trials comparing antiplatelet and anticoagulant therapy in patients with embolic stroke of undetermined source have demonstrated no superiority of anticoagulation in preventing recurrent ischemic stroke and have suggested an increased risk of bleeding; therefore, routine anticoagulation is not recommended in this population [8,9]. Consequently, thorough investigation of the embolic source is essential for guiding appropriate antithrombotic therapy. In this case, extending the field of view of contrast-enhanced CT at stroke onset to include the entire trunk might have been useful for earlier detection or exclusion of venous thrombi.

Histopathological examination of emboli retrieved by mechanical thrombectomy has been increasingly reported to contribute to the identification of rare causes of cerebral embolism, highlighting the importance of analyzing retrieved thrombi [10,11]. A recent study analyzing thrombus composition has suggested that thrombi associated with patent foramen ovale–related cerebral embolism tend to have a relatively low red blood cell content and are predominantly composed of fibrin and platelets [12]. These findings support the hypothesis that a patent foramen ovale may contribute not only as a conduit for thrombus passage but also as a site of thrombus modification or formation [1,12]. Although paradoxical embolism has traditionally been attributed to red blood cell–rich venous thrombi passing through a patent foramen ovale, the composition of venous thrombi and cerebral emboli frequently differs. Anatomically, a patent foramen ovale forms a slit-like tunnel structure, through which venous thrombi may undergo fragmentation, reorganization, and rethrombosis, resulting in alterations in thrombus composition [12,13]. During this process, softer red blood cell–rich components may be lost, whereas relatively rigid fibrin- and platelet-rich components may be retained. Although histopathological findings alone were insufficient to establish a definitive diagnosis of paradoxical embolism, analysis of retrieved emboli may provide important clues when the embolic source remains unclear. However, thrombus composition is not specific to paradoxical embolism and does not allow direct inference of the embolic pathway; therefore, these findings should be interpreted with caution.

Percutaneous closure of a patent foramen ovale is considered in patients younger than 60 years with embolic stroke of undetermined source when the patent foramen ovale is strongly suspected to be causative [14]. High-risk features associated with greater benefit from patent foramen ovale closure include the presence of a large right-to-left shunt, defined as a shunt present at rest or extensive microbubble passage during the Valsalva maneuver, and concomitant atrial septal aneurysm with a septal excursion of 15 mm or greater [15]. In the present case, the shunt markedly increased during the Valsalva maneuver, and an atrial septal aneurysm was also present, indicating a functionally significant and high-risk patent foramen ovale. Although percutaneous closure was considered, it was not performed because of the patient’s nickel allergy.

The mechanisms of venous thrombus formation are classically described by Virchow’s triad: venous stasis, endothelial injury, and hypercoagulability [16]. Venous stasis may result from prolonged immobilization or sitting, heart failure, lower-extremity varicosities, pregnancy, or the postpartum period. Endothelial injury may be caused by trauma, iatrogenic factors such as medical procedures or medications, infection, or inflammatory diseases. Hypercoagulability can be congenital or acquired, including malignancy, antiphospholipid syndrome, pregnancy, oral contraceptive use, obesity, smoking, dehydration, and inflammatory conditions. In the present case, smoking was the only identifiable risk factor for venous thrombosis, which alone was insufficient to fully explain thrombus formation. Because long-term anticoagulation therapy is required, further evaluation of the underlying causes of venous thrombosis remains important.

This case highlights the importance of comprehensive imaging, including evaluation of the pulmonary arteries, when the embolic source remains unclear, and may aid diagnostic reasoning and treatment selection in similar clinical scenarios.

## Conclusion

Even in the absence of detectable deep vein thrombosis, identification of a pulmonary artery thrombus may provide important diagnostic clues and support the diagnosis of paradoxical embolism.

## Declaration of generative AI and AI-assisted technologies in the manuscript preparation process

During the preparation of this work, the authors used ChatGPT (OpenAI) to assist with language editing and refinement of the manuscript. After using this tool, the authors reviewed and edited the content as needed and take full responsibility for the content of the published article.

## Author contributions

AI and HI contributed to study conception, data collection, and manuscript drafting. MO, TI, HC, ST, NS, TK, and CS contributed to clinical management and critical revision of the manuscript. YA supervised the study. All authors read and approved the final manuscript.

## Patient consent

Written informed consent was obtained from the patient for publication of this case report and any accompanying images.
